# Progress in Otology

**Published:** 1896-07-04

**Authors:** 


### Progress in Otology.

**Tuberculous Disease of the Middle Ear.**—Dr. Milligan[1] refers in this paper to primary tubercular affections of the middle ear amongst patients apparently otherwise in good health. The paths along which the tubercle bacillus may reach the cavum tympani are: (1) Along the Eustachian tube; (2) along the blood vessels and channels of the tympanic mucosa; (3) through an already existing perforation of the membrana tympani. Once the bacillus has gained a foothold in the tympanum, the further progress of disease set up by it may be acute or chronic. In the acute form the mucous membrane rapidly becomes infiltrated and degenerated, bacilli being found at times in considerable numbers in the discharge, though, in all tubercular cases, their discovery is a matter of difficulty and uncertainty. In the more chronic forms, miliary tubercles at times visible to the naked eye are deposited within the mucous membrane; these soon caseate and ulcerate. In favourable cases fibrous tissue formation puts an end to the process, but far more commonly the disease extends to the underlying bone, and cario-necrosis is set up, which implicates the labyrinth, mastoid, or intracranial cavity. The insidious character of tuberculous inflammation of the middle ear is well known, but Milligan points out that in rare cases extensive destruction of this region may take place without any perforation of the membrana tympani occurring. In such cases the first symptoms noticed beyond the deterioration of the general health are either enlargement of the glands around the ear, or the sudden occurrence of facial paralysis. No one symptom or set of symptoms, however, can be regarded as pathognomonic of tubercle, the inoculation test can alone establish the diagnosis beyond a doubt, and this within the comparatively short period of from two to four weeks. Of ten cases clinically considered by Milligan to be tubercular, he established the diagnosis by inoculation in eight.

The progress of the inflammatory process in tuberculous disease is in strong contrast with the sthenic and simple inflammatory type. A thin purulent fluid forms gradually in the middle ear without pain or rise of temperature, and the membrana tympani melts slowly away, while the edges of the perforation are found uninflamed and sodden in appearance. Cicatrization is extremely rare; the bone becomes early and extensively involved. The prognosis is grave, especially in young patients, and as regards treatment, free drainage should be early established from the middle ear by timely operation upon the mastoid; this gives the patient the best chance of life and of retaining the greatest amount of hearing power. Soft and disintegrating foci of bone must be thoroughly eradicated, or a recurrence of the disease is sure to take place. The wound is best treated on the "dry" principle. The general health must be perseveringly treated by those rules of diet, medicine, and hygiene which are applicable to all subjects of the tuberculous diathesis.

**Rupture of the Drumhead by Blows upon the Ear.**—In a clinical lecture upon three cases of this injury, Barclay[2] describes his method of securing coaptation of the lips of the perforation when sufficiently small, by means of the "Blake paper disk" or splint. This consists of a circular piece cut from sized writing paper, and of sufficient diameter to overlap the perforation. H.ving been previously moistened with sterilized lotion, the disk is carried on the end of a probe tipped with cotton wool to the perforation, to which it is applied concentrically, pressed very lightly with a dry cotton-wool brush or probe into "snug" apposition with the lips of the wound, and left there. Within a day or two it will become cemented in its place by the sizing on the paper and by exudation, and in about a week's time it may be removed and the perforation will firmly close. The disk appears not to be suited for valvular or circular perforations until, at all events, they have partially closed under ordinary treatment, when, after a few days, it may be applied with advantage.

**The Stacke Operation for the Cure of Chronic Otorrhœa.**—Although an operation now frequently practised, this is one of which but few text books, even the most recent, give an account, and the published literature on the subject is sparse, at least in our own language. Indeed, Bates, when alluding in the present article to Holmes' description with twelve cases in the Arch. of Otol., 1893, No. 4, refers us to practically the only available paper, although the operation was proposed so long ago as 1891. Bates describes the steps of the operation as he has recently performed it, as follows: An incision is made over the mastoid, close to the insertion of the auricle, and extending from its upper margin to the tip of the mastoid. The tissues are divided down to the bone, and the auricle dissected from it as far forward as the meatus. The membranous meatus is cut across at its attachment to the bone, and the auricle drawn forward with a blunt retractor. The posterior wall of the meatus is then removed by the chisel as far inward as the plane of the posterior insertion of the membrana tympani. When the membrane is absent, a bent probe or strabismus hook is inserted in the aditus and employed as a guide to indicate the depth to which the posterior wall of the meatus could be chiselled without injury to the facial nerve. The next point is the opening up by the shortest route of the aditus, which, like the antrum, is always (according to Politzer) diseased in chronic suppuration of the tympanum, and this may be done by removing the bone external to it. The facial canal lies very superficially in its inner wall, and requires to be carefully avoided. The outer wall of the aditus being removed, the promontory is usually seen, and also, less readily, the ridge formed by the canal for the facial nerve. The greatest care is necessary in chiselling, to avoid injury to the latter, which courses along close to the posterior wall of the tympanum, and curves upward and forward skirting its superior margin. The antrum is now opened without difficulty, and the outer wall of the attic having been already removed, the tympanum is cleared by removing the remnants of the membrane malleus and incus, leaving the stapes, and curetting the bone till it is smooth. The success of the operation depends largely upon the thorough removal of all necrotic tissue. Bony sinuses must be searched for and laid open, removing more of the outer table of the mastoid, if necessary. When the chiselling is completed, the canal, antrum, attic, aditus, and tympanum are converted into one large irregular cavity. Bates made no attempt to carpet the denuded bone with flaps derived from the membranous meatus, as suggested by Stacke, owing to its impracticability, in his cases. The wound behind the auricle was closed by interrupted silk sutures, and a cotton collodion dressing applied over it, after dusting with iodoform. The only new feature in the after treatment seems to have consisted in the instillation of balsam of Peru into the meatus several times a day. This is said to have prevented infection, for in the cases not so treated the discharge became purulent, and the healing process was delayed. Out of his ten cases, Bates had only one death. In three, facial paralysis from "preventable causes" occurred, but all these improved. The hearing was not improved by the operation, but it was considerably in many cases by the after treatment. Bates concludes that the Stacke operation is an improvement upon the "classical mastoid" operation, and should always be done in acute and chronic mastoiditis when there has been suppuration of the tympanic cavity.

**Cochlear Apoplexy.**—Cases of sudden deafness due to effusions into the labyrinth are fairly common, but this case described by Dr. Milligan[4] is one of special interest, on account of the succinct manner in which it has been reported, and the possibility it afforded of localising very precisely the region of the internal ear affected.

A gentleman aged 41, with good previous history, but a very high liver, and with a consequently high arterial tension, and dilated cutaneous venules, retired to bed in his usual health. Early in the morning he woke up with a loud noise in his head, and perfectly deaf in his left ear; he had no giddiness, no sickness, and no pain; no "Menière's complex of symptoms" in fact, and he could talk and write correctly. The lower tones of the tuning fork were audible, but high-pitched ones and Galton's whistle were not heard. The result of treatment was entirely negative, and the prognosis (justified by results) was bad. In the discussion following this paper, the diagnosis was fully supported, and further refined by Dundas Grant, who suggested that owing to the well-known fact that the lower whorl of the cochlea was most affected by increased blood pressure, the effusion of blood might safely be located there, and the semi-circular canals for very good reasons excluded.

**"Morbus Menierei."**—Professor Grüber has recently attempted, in a paper read before the Austrian Otological Society,[5] to banish the term "Menière's symptoms" from our nosology and retain only that of "Menière's disease," and for the conditions to which Menière originally applied it, viz., primary labyrinthine affections. He argued that, owing to the varying anatomical formation of the saccus endolymphaticus and of the aqueductus vestibuli, which may be exceptionally small, the outflow of the endolymph may easily be interfered with, giving rise to vertigo and timitus. Hence, being common complications of disease of the middle ear, Grüber considered that these symptoms should not constitute the main term in the formal diagnosis of the case. The suggestion did not, however, meet with practical support in the discussion.

**Transplantation of Thiersch's Skin Grafts in Stacke's Operation.**—Politzer demonstrated before the same society[6] the case of a woman, aged 46, in whom he had adopted this method. The radical operation for removal of cholesteatomatous masses in the cavum and attic, and laying open a fistulous track extending from the tip of the mastoid to the inferior wall of the tympanic cavity, had been carried out. Six days later Thiersch's transplantation was performed with flaps of skin taken from the left forearm. On the anterior and upper wall of the wound the grafts succeeded perfectly, but the new skin did not adhere to the posterior wall. Sufficient skinning over took place to prevent adhesions of the walls of the passage. Nevertheless, it was decided to carry out fresh grafts on the posterior wall.

**Exostosis of the External Meatus.**[7]—Politzer has recently had an opportunity of dissecting a rare form of this disease which had been under his observation for thirty years, and died without the patient having consented to operation. The exostosis was of irregular surface, 1½ cm. in length and 1 cm. in breadth, and grew from the postero-superior margin of the meatal orifice. The lumen of the meatus was occupied by a brownish-yellow epidermic mass extending into the tympanum through a small orifice in the membrane. When this was removed the deep extremity of the meatus was found to be considerably dilated, and its superior wall corroded from pressure. A small orifice existed close to Schrapnell's membrane, leading to a cavity the size of a cherrystone, and this communicated directly with the mastoid antrum. The retention of epidermic masses furnished an explanation for these osseous defects, the exostosis itself being probably due to a previous otorrhœa.

**Iodoform Gauze Plugging of the Meatus in Inflammatory and Suppurative Affections of the Middle Ear.**[8]—Hamon du Fougeray practises this method, which is founded on the views of Lermoyez and Helme, as already reported in this journal. The idea is to prevent (1) access of aerial germs and (2) auto-inoculation. A strip of iodoform gauze of three or four thicknesses is cut so as to have a length of three cm. and the width of one. This is introduced into the meatus as far as, but not touching the drum, and reaching up to the orifice. The patient is then left until the next dressing. When the suppuration is abundant the gauze must be renewed every twenty-four hours, but it may be left if otherwise as long as three or four days, and should be continued for some time after the discharge has ceased. The result, as tested in eighty cases of all forms of external and median otitic suppuration, was extremely satisfactory.

[1] Med. Chron., Jan., 1896. [2] Internat. Clin., iv., 2. [3] Amer. Med. Surg. Bull., Jan. 18, 1896. [4] Med. Chron., Jan., 1896. [5] Jnal. of Lar., Mar. 18, 1896. [6] Ibid. [7] Int. Clin., iv., 3. [8] Proc. French Soc. of Lar. and Otol., reported in Jnal. of Lar., Feb., 1896.

